# Epigenetic modification of m^6^A regulator proteins in cancer

**DOI:** 10.1186/s12943-023-01810-1

**Published:** 2023-06-30

**Authors:** Yumin Wang, Yan Wang, Harsh Patel, Jichao Chen, Jinhua Wang, Zhe-Sheng Chen, Hongquan Wang

**Affiliations:** 1 Department of Respiratory and Critical Care Medicine, Aerospace Center Hospital, Peking University Aerospace School of Clinical Medicine, Beijing, 100049 China; 2 Hunan Provincial Key Laboratory of Hepatobiliary Disease Research, Division of Hepato-Biliary-Pancreatic Surgery, Department of Surgery, The Second Xiangya Hospital of Central South University, Changsha, 410008, China; 3 Department of Pharmaceutical Sciences, College of Pharmacy and Health Sciences, St. John’s University, Queens, NY 11439 USA; 4 Beijing Key Laboratory of Drug Target and Screening Research, Institute of Materia Medica, Chinese Academy of Medical Sciences and Peking Union Medical College, Beijing, 100050 China; 5 Department of Pancreatic Cancer, Tianjin Medical University Cancer Institute and Hospital, National Clinical Research Center for Cancer, Tianjin’s Clinical Research Center for Cancer, Key Laboratory of Cancer Prevention and Therapy, Tianjin, 300060 China

**Keywords:** Cancer, N_6_-methyladenosine methylation, RNA modification, m^6^A regulators, m^6^A methylation enzymes

## Abstract

Divergent N_6_-methyladenosine (m^6^A) modifications are dynamic and reversible posttranscriptional RNA modifications that are mediated by m^6^A regulators or m^6^A RNA methylation regulators, i.e., methyltransferases (“writers”), demethylases (“erasers”), and m^6^A-binding proteins (“readers”). Aberrant m^6^A modifications are associated with cancer occurrence, development, progression, and prognosis. Numerous studies have established that aberrant m^6^A regulators function as either tumor suppressors or oncogenes in multiple tumor types. However, the functions and mechanisms of m^6^A regulators in cancer remain largely elusive and should be explored. Emerging studies suggest that m^6^A regulators can be modulated by epigenetic modifications, namely, ubiquitination, SUMOylation, acetylation, methylation, phosphorylation, O-GlcNAcylation, ISGylation, and lactylation or via noncoding RNA action, in cancer. This review summarizes the current roles of m^6^A regulators in cancer. The roles and mechanisms for epigenetic modification of m^6^A regulators in cancer genesis are segregated. The review will improve the understanding of the epigenetic regulatory mechanisms of m^6^A regulators.

## Background

**Fig. 1:**
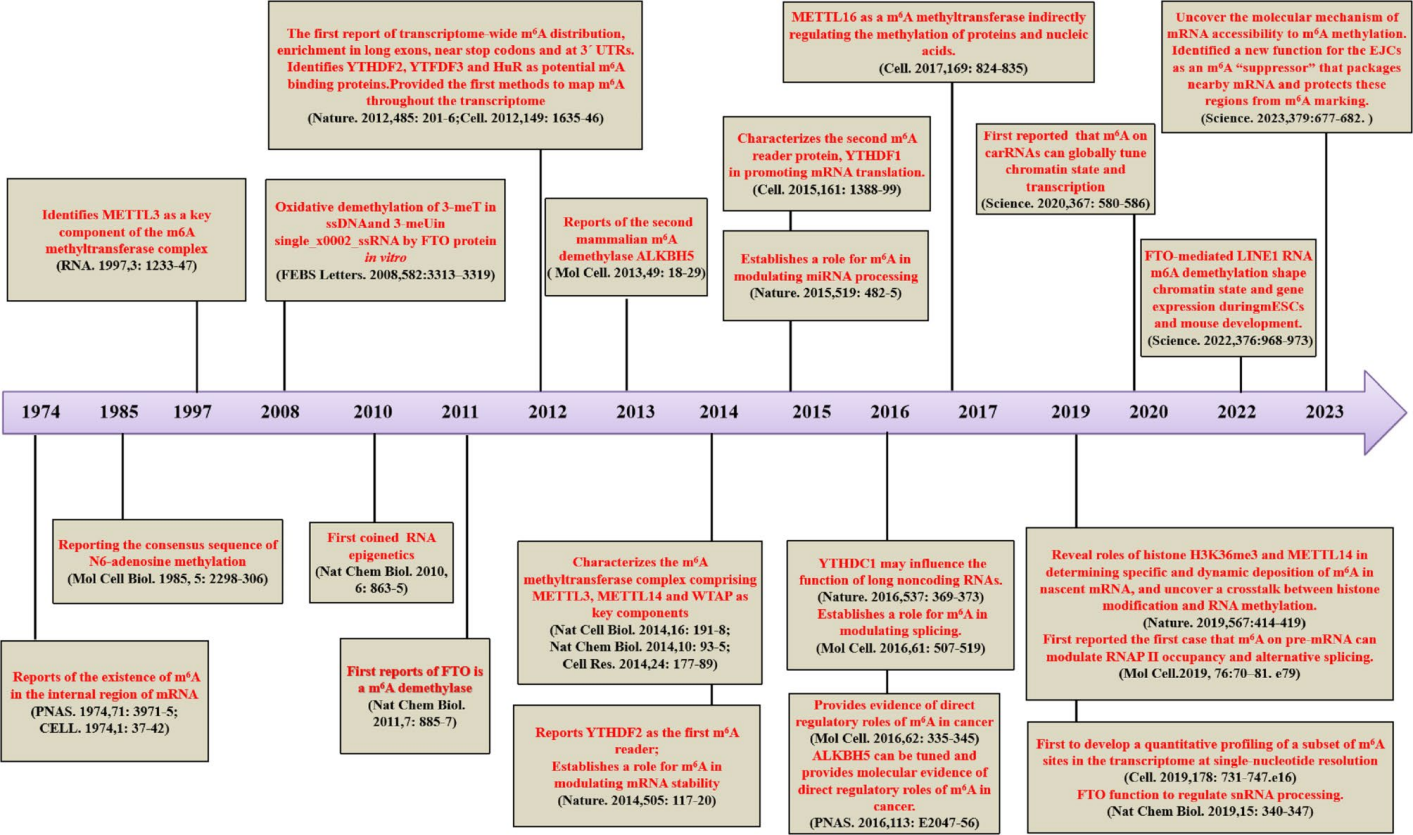
Timeline diagram depicting essential discoveries in the field of m^6^A research

**Fig. 2:**
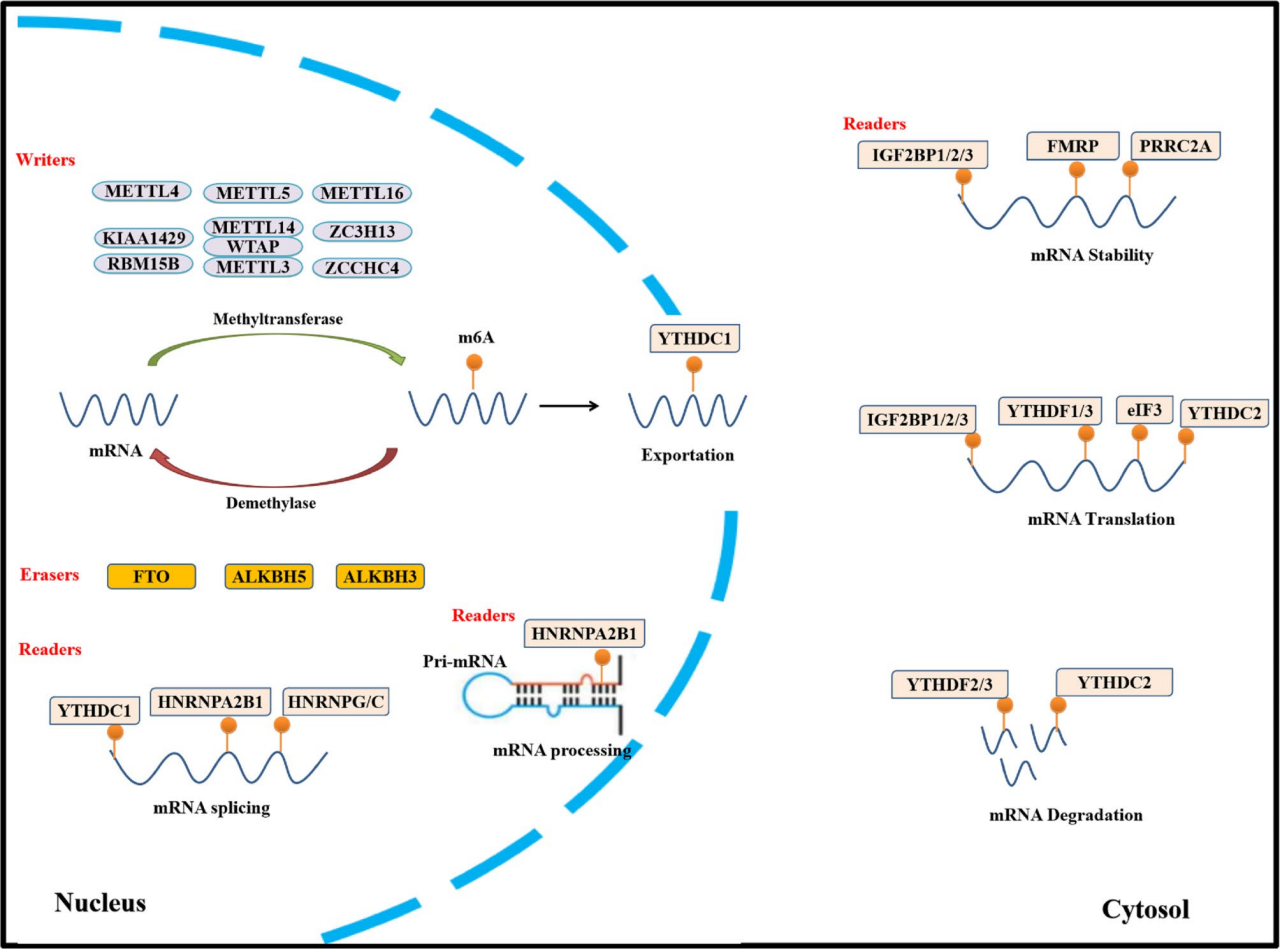
m^6^A regulator proteins and the underlying mechanisms of m^6^A modification. The m^6^A modification of mRNA is mainly catalyzed by the core methylase complex METTL3-WTAP-METTL14. RBM15/15B, VIRMA/KIAA1429, and ZC3H13 are newly identified mRNA modification writers; METTL4, and METTL16 are snRNA modification writhers; and METTL5 and ZCCHC4 are rRNA m^6^A writers. The m^6^A modification is removed by FTO, ALKBH5, and ALKBH3. Readers that include members of the YTH domain-containing family, the IGF2BP family, the HNRNP family, eIF3, PRRC2A, and FMRP, recognize modification and affect various functions of RNAs

Similar to DNA and proteins, RNA can undergo more than 170 post-transcriptional modifications [1]. In the 1970s, adenosine, an RNA building block, was demonstrated to be methylated at N^6^ nitrogen atom (i.e., N^6^-methyladenosine (m^6^A) formation) [2, 3]. Consequently, m^6^A modification has been identified as the most abundant cellular modification in mammalian mRNA. A pioneer study demonstrated, for the first time, role of m6A in mRNA stability [4], followed by cloning and discovery (in 1997) of methyltransferase-like protein 3 (METTL3), which synthesizes nearly all m^6^A in the mRNA transcriptome (Fig. 1) [5]. In addition, other studies have shown that m^6^A is essential for regulation of many developmental processes [6, 7]. This has resulted in rapid development of detection and transcriptome-wide mapping technologies for m^6^A-containing transcripts, enabling detection in nearly all types of RNAs, including mRNAs, small nuclear RNAs (snRNAs), ribosomal RNAs (rRNAs), and several species of regulatory RNAs [8]. Previous studies have largely focused on delineating the role of m^6^A methylation in mRNA metabolism and tumor progression; however, emerging evidence has revealed that m^6^A is involved in almost all RNA metabolic processes, such as mRNA maturation, transcription, translation, degradation, and stability. Dysregulation of m^6^A results in pathogenesis of multiple human diseases, including cancer. Growing evidence suggests m^6^A alteration is involved in tumorigenesis through many regulatory mechanisms in programmed cell death [9], metabolism [10], drug resistance [11], oncogene and/or tumor suppressor expression [12], immunotherapy [13], and targeted therapy [14]. The m^6^A RNA modification is dynamically and reversibly regulated by three enzymes, namely, m^6^A methyltransferases (“writers”), m^6^A demethylases (“erasers”), and m^6^A binding proteins (“readers”), that establish a complex interplay between m^6^A incorporation, degradation, and recognition [15, 16]. Enzymes mediating m^6^A effects are defined as m^6^A regulators or m^6^A RNA methylation regulators (Fig. 2) [14, 16]. Methyltransferases install m^6^A, demethylases remove m^6^A, and m^6^A-binding proteins recognize and act upon m6A-modified RNA. While writers and erasers determine the distribution and prevalence of m^6^A, readers mediate m^6^A-dependent functions [16]. Accumulating evidence has revealed that writers, erasers, and readers are frequently disordered and are involved in cancer pathogenesis by regulating the expression of oncogenes and/or tumor suppressors, promoting cancer proliferation, development, metastasis, and tumorigenesis [10–12, 14, 17, 18]. While previous studies mostly focused on the role of m^6^A RNA methylation in tumorigenesis, recent studies have explored m^6^A regulators in cancer genesis. Nevertheless, the functions and mechanisms of m^6^A regulators are unknown and need to be elucidated in cancer. Since 2015 [19], studies have revealed that m^6^A regulatory proteins are regulated by epigenetic modifications, such as ubiquitination, SUMOylation, acetylation, methylation, phosphorylation, and lactylation, or via noncoding RNA action, in cancer. In this review, a concise overview of the current understanding of the role of m^6^A regulators in cancer is provided. Additionally, the roles and mechanisms of epigenetic modifications of m^6^A regulators in cancer genesis are delineated. This review will enhance the understanding of the epigenetic regulatory mechanisms of m^6^A regulators.

## m^6^A regulator proteins: m^6^A writers, erasers, and readers

The m^6^A writers, erasers, and readers constitute the molecular composition of m^6^A RNA methylation regulator proteins [14]. These are proteins that insert (writers), remove (erasers), and recognize (readers) m^6^A on mRNAs or noncoding RNAs. Proteins that mediate the effects of m^6^A establish a complex interplay between the above three m^6^A functions [15]. The effects of m^6^A on mRNA expression are mediated by an expanding list of m^6^A readers and m^6^A writer-complex components, as well as potential erasers. The mechanisms and effects of m^6^A-modifying regulatory proteins on RNA metabolism are summarized in Table 1.

**Table 1:** The function of m^6^A modifcation regulators (m^6^A methylation enzymes) in RNA metabolism

Types	m^6^A Regulator	Full names	Cellular localization	Function	Ref
Writers	METTL3	Methyltransferase-like 3	Nucleus	Catalyzes methylation reaction/Catalyzes m6A modifcation	[5, 20, 21]
	WTAP	Wilms tumor 1- associated protein	Nucleus	Promotes METTL3-METTL14 heterodimer localization into nuclear speckles	[22, 23]
	METTL14	Methyltransferase-like 14	Nucleus	Assists METTL3 to recognize the subtract	[20, 22]
	VIRMA (KIAA1429)	Vir-like m6A methyltransferase associated	Nucleus	Recruits the m6A complex to the special RNA site and interacts with polyadenylation cleavage factors CPSF5 and CPSF6	[24, 25]
	RBM15	RNA binding motif protein 15	Nucleus	Directs METTL3-METTL14 heterodimer to specifc RNA sites	[24, 26]
	RBM15B	RNA binding motif protein 15B	Nucleus	Directs METTL3-METTL14 heterodimer to specifc RNA sites	
	METTL16	Methyltransferase-like 16	Nucleus	Catalyzes m6 A modifcation; mediate the m6A methylation of U6 snRNA, noncoding RNAs, and precursor mRNAs (premRNAs)	[27–29]
	ZC3H13	Zinc finger CCCH-type containing 13	Nucleus	Bridges WTAP to the mRNA-binding factor Nito;Anchors WTAP in the nucleus to enhance m6A modifcation	[30, 31]
	METTL5	Methyltransferase-like 5	Nucleus	Induce the m6A methylation of 18 S rRNA	[32]
	ZCCHC4	Zinc finger CCHC-type containing 4	Nucleus	An m6A methyltransferase of 28 S rRNA mediating ribosomal RNA methylation	[33–35]
	METTL4	Methyltransferase-like 4	Nucleus	Mediates the m6A methylation of U2 snRNA to regulate pre-mRNA splicing	[36]
Erasers	FTO	Fat mass and obesity -associated protein	Nucleus	Acts as m6A demethylase to promote mRNA splicing and translation; removes m6A modification	[37]
	ALKBH5	AlkB homologue 5	Nucleus	Removes m6A modifcation to promote mRNA nuclear processing and mRNA export	[38]
	ALKBH3	AlkB homologue 3	Nucleus	Remove m6 A modifcation level	[39]
Readers	YTHDF2	YTH N6-methyladenosine RNA binding protein 2	Cytosol	Promotes mRNA degradation	[40, 41]
	YTHDF1	YTH N6-methyladenosine RNA binding protein 1	Cytosol	Promotes mRNA translation initiation	[42]
	eIF3	Eukaryotic translation initiation factor 3 subunit A	Cytosol	Promotes mRNA translation	[43]
	HNRNPA2B1	Heterogeneous nuclear ribonucleoprotein A2/B1	Nucleus	Promotes primary miRNA processing and mRNA splicing; promotes primary microRNA processing and mediates nuclear accumulation	[44]
	HNRNPC	Heterogeneous nuclear ribonucleoprotein C	Nucleus	Mediates mRNA splicing and maturity Interacts with m6A-modifed mRNA and affects its enrichment and splicing, generating a phenomenon termed the “m6A switch”	[45, 46]
	HNRNPG	Heterogeneous nuclear ribonucleoprotein G	Nucleus	Mediates mRNA splicing and maturity	[45, 46]
	YTHDC1	YTH domain containing 1	Nucleus	Promotes mRNA splicing and transcriptional silencing; regulates RNA nuclear export and splicing	[47, 48]
	YTHDF3	YTH N6-methyladenosine RNA binding protein 3	Cytosol	Interacts with YTHDF1 to promote mRNA translation or interacts with YTHDF2 to promote mRNA degradation	[49, 50]
	YTHDC2	YTH domain containing 2	Nucleus; cytosol	Improves the translation efciency of target mRNA	[51]
	IGF2BP1	Insulin-like growth factor 2 mRNA binding protein 1	Nucleus; cytosol	Promotes the stability and translation of mRNA	[52]
	IGF2BP2	Insulin-like growth factor 2 mRNA binding protein 2	Nucleus; cytosol	Promotes the stability and translation of mRNA	[52]
	IGF2BP3	Insulin-like growth factor 2 mRNA binding protein 3	Nucleus; cytosol	Promotes the stability and translation of mRNA	[52]
	FMRP	Fragile X mental retardation protein	Nucleus; cytosol	Promote the nuclear export and stability of m6A-modifed RNAs	[53, 54]
	PRRC2A	Proline rich coiled-coil 2 A	Cytosol	Bind to a consensus GGACU motif in the Olig2 coding sequence to stabilize Olig2 mRNA	[46]
	RBM33	RNA-binding motif protein 33	Nucleus	Forms a complex with ALKBH5 and mediates m6 A demethylation of selected transcripts by regulating ALKBH5 substrate accessibility and activity	[55]

## Writers

The currently known m^6^A methyltransferases, or “m6A writers”, include methyltransferase-like 3 (METTL3), methyltransferase-like 14 (METTL14), wilms tumor 1-associated protein (WTAP), RNA binding motif protein 15/15B (RBM15/RBM15B), vir-like m6A methyltransferase associated (VIRMA or KIAA1429), zinc finger CCCH-type containing 13 (ZC3H13), methyltransferase-like 16 (METTL16), methyltransferase-like 4 (METTL4), methyltransferase-like 5 (METTL5), and zinc finger CCHC-type containing 4 (ZCCHC4) (Table 1). The m^6^A, first reported om 1994, is a multicomponent methyltransferase complex [56]. Subsequently, METTL3, an S-adenosyl-methionine-binding protein with methyltransferase activity, was identified [5]. Recent studies have identified additional components of the m^6^A methyltransferase complex in mammals, namely, METTL14 [22, 57] and WTAP [22, 23], which are known to form a complex with METTL3 and are anchored to the nucleus to catalyze m^6^A methyltransferases [22, 23]. While **METTL3** functions as a key catalytic component of the m^6^A methyltransferase complex [5], **METTL14** is the core subunit of m^6^A methyltransferase for m^6^A installation [22] and **WTAP** is the regulatory subunit of m^6^A methyltransferase facilitating m^6^A modification [22, 23]. **RBM15/15B** is a subunit of the writer complex and facilitates the recruitment of the m^6^A writer complex to RNA by interacting with METTL3 in a WTAP-dependent manner [26, 58]. **VIRMA** (originally known as KIAA1429) is a regulatory subunit of m^6^A methyltransferase that facilitates m^6^A installation and functions as a WTAP interactor to associate with the METTL3/METTL14/WTAP complex, coordinatively modulating m^6^A modification [25, 58]. The ablation of VIRMA leads to a substantial loss of m^6^A in *D. melanogaster* [59] and mammalian cells [24]. VIRMA recruits the m6A complex to specific RNA sites and interacts with the polyadenylation cleavage factors CPSF5 and CPSF6, resulting in prolonged 3ʹUTR selection [25]. **ZC3H13** interacts with WTAP and anchors it in the nucleus to promote m^6^A modification [31, 58], facilitating m6A addition and stem cell renewal [31]. Deletion of ZC3H13 resulted in the loss of m^6^A in *D. melanogaster* [30, 60] and approximately 80% loss of cellular m^6^A in mammalian cells [30], suggesting that some m^6^A sites are formed independent of ZC3H13. Similar to WTAP, ZC3H13 is important for the nuclear localization of the writer complex [31] and is assumed to promote RBM15/15B interaction with WTAP to facilitate methylation [30]. **METTL16** mediates the insertion of m^6^A in small nuclear RNA (snRNAs) (e.g., the spliceosome component U6 snRNA) [27, 29]. METTL16 also functions as a methyltransferase and catalyzes m^6^A addition in U6-like sequences of MAT2A mRNA, the enzyme required for the biosynthesis of S-adenosylmethionine (SAM) [27, 61]. In addition, METTL16 catalyzes the addition of m^6^A in a small number of noncoding RNAs and mRNAs [29]. **ZCCHC4** is a ribosomal RNA (rRNA)-adenosine-methyltransferase responsible for the formation of a single m^6^A residue in the 28 S ribosomal RNA (rRNA) [32, 62]. The addition of m^6^A on unique, highly conserved sites in the 18 S rRNA of eukaryotes is mediated by **METTL5**-TRMT112 complex, in which METTL5 functions as the catalytic subunit and TRMT112 as an allosteric adaptor [32]. **METTL4** mediates m^6^A methylation of U2 snRNAs to regulate pre-mRNA splicing [36, 63].

## Erasers

The m^6^A incorporation and removal in mRNA is a dynamic and reversible process, confirmed in 2011 with the discovery of the fat mass and obesity-associated protein (FTO), which is the first m^6^A demethylase that removes the methyl group to restore the methylated base to the adenine base [37]. **FTO** displays m^6^A demethylase activity and demethylates m^6^A residues in mRNA indicating the reversibility of this modification [37]. Mauer et al. characterized FTO as a m^6^A demethylase that regulates mRNA stability and suggested that m^6^A is a dynamic reversible modification, rekindling interest in the biological relevance of m^6^A [64]. Furthermore, Zheng et al. discovered the second mammalian m^6^A demethylase, namely, alkB homologue 5 (**ALKBH5**), that affects mouse spermatogenesis and demonstrated that m^6^A is a dynamic reversible modification of mRNA [38]. FTO and ALKBH5 facilitate the removal of m^6^A and potentially affect different subsets of target mRNAs because of their distinct subcellular and tissue distributions [37, 38]. The first evidence of reversible post-transcriptional modification was given when FTO and ALKBH5 removed addition of m^6^A in mRNA and certain noncoding RNAs transcribed by RNA polymerase II [37, 38]. By definition, ALKBH3 is an eraser responsible for the removal of the m^6^A modification on the tRNA [39].

## Readers

m^6^A can recruit m^6^A-binding proteins or m^6^A readers that mediate m^6^A-dependent functions to regulate the fate of mRNAs [16, 65]. The m^6^A readers regulate mRNA nuclear export, splicing, degradation, translation, and stability. The first discovered m^6^A reader family, providing a mechanistic basis for understanding the effects of m^6^A on mRNA, was the YT521-B homology (YTH) domain family of proteins [66]. The YTH domain family includes YTHDF1, YTHDF2, YTHDF3, YTHDC1, and YTHDC2. The nuclear m^6^A readers are YTHDC1, HNRNPC11, HNRNPA2B1, and HNRNPG, whereas m^6^A readers in the cytosol are YTHDC2, YTHDF1/2/3, and IGF2BP1/2/3. Different readers have different m^6^A positioning functions [67]. **YTHDF2**, the first discovered m^6^A-binding protein, regulates mRNA degradation by mediating the lifetime of target transcripts [41, 66]. Similarly, **YTHDF1** promotes translation of m^6^A-modified mRNAs in the cytosol [42], while **YTHDF3** cooperates with YTHDF1 and YTHDF2 to modulate the translation and degradation of m^6^A-labelled mRNA and inversely regulates their functions [50]. The insulin-like growth factor 2 mRNA-binding proteins (**IGF2BP1/2/3**) promote mRNA stability and translation [52]. **FMRP** enhances the nuclear export and stability of m^6^A-decorated RNAs [53, 54]. Furthermore, **YTHDC1** modulates nuclear export and splicing of m6A-modified RNAs [47, 48], while **YTHDC2** regulates the translation and abundance of target genes [51]. As a multiprotein complex that recruits small ribosomal subunits to mRNAs, **Eukaryotic initiation factor 3 (eIF3)** preferentially binds to m^6^A-decorated mRNA and is involved in mRNA translation [42, 68]. YTHDF1 recruits eIF3 to the 5’ end of the transcripts, resulting in YTHDF1 looping that modulates initiation of translation [42]. The heterogeneous nuclear ribonucleoprotein (HNRNP) proteins include HNRNPA2B1, HNRNPC, and HNRNPG. HNRNPA2B1 [44] and HNRNPC[45] are active splicing regulators that can selectively bind m^6^A-decorated mRNAs [45, 69, 70]. **HNRNPA2B1** recognizes m^6^A-labelled primary miRNAs (pri-miRNAs) and regulates alternative splicing events [44] and miRNA biogenesis [44, 71]. **HNRNPC** recognizes m^6^A-induced changes in secondary mRNA structures [45], and **HNRNPG** is an RNA-binding protein involved in the splicing of m^6^A-labelled mRNA[72]. Proline-rich coiled-coil 2 A (PRRC2A) was later identified as a novel m^6^A reader that binds to a consensus GGACU motif in the Olig2 coding sequence to stabilize Olig2 mRNA [46].

## m^6^A regulator proteins and cancer

Previous studies have shown that m^6^A is associated with numerous human diseases, including cancer. Pioneering studies have provided molecular evidence of the direct regulatory roles of m^6^A in cancer [73, 74]. The ablation of METTL3 caused apoptosis and reduced the invasiveness of lung adenocarcinoma cells [73], whereas hypoxia-activated m^6^A demethylase ALKBH5 induces the accumulation of breast cancer stem cells through HIF-dependent and ALKBH5-mediated m^6^A demethylation of NANOG mRNA [74]. Recent evidence has indicated that m^6^A regulatory proteins, i.e., writers, erasers, and readers, play a role in various types of human cancers by contributing to malignancy. This includes cancer cell proliferation, self-renewal of cancer stem cells, and resistance to radiotherapy or chemotherapy. Comprehensive reviews for detailed discussions on the role of m^6^A regulatory proteins in cancer are already available in literature [11, 12, 14, 17, 18, 67, 75–79]. However, the functions and mechanisms of m^6^A regulators in cancer remain largely unestablished and need future investigations.

## Epigenetic modification of m^6^A regulators and tumorigenesis

Epigenetics is a reversible and dynamic process that regulates gene expression without altering DNA. There are four major mechanisms of epigenetic regulation: DNA methylation, histone modification, chromatin structure regulation, and noncoding RNA regulation [80, 81]. All mechanisms, except chromatin structure regulation, have been studied extensively [82]. The histone subunit in the nucleosome possesses a characteristic tail containing specific amino acids for covalent posttranslational modifications (PTMs), such as acetylation, methylation, ubiquitylation, phosphorylation, glycosylation, sumoylation, acylation, glycation, hydroxylation, serotonylation, and ADP-ribosylation [83–86]. Recent studies have suggested that m^6^A regulators in cancer can be modulated by epigenetic modifications, including ubiquitination, SUMOylation, acetylation, lactylation, O-GlcNAcylation, methylation, phosphorylation, ISGylation, and noncoding RNA. Hence, this section focuses on the roles and mechanisms of the epigenetic modification of m^6^A regulators in cancer genesis. The effects and mechanisms of epigenetic modification of m^6^A regulatory proteins in tumorigenesis are summarized in Table 2.

**Table 2:** Epigenetic modification of m^6^A Regulator proteins in tumorigenesis

Modification	m^6^A Regulator	Cancer	Involved mechanism	Ref
Ubiquitination	FTO	CRC	GSK3β mediated ubiquitination of demethylase FTO to reduce FTO expression. GSK3β suppresses the progression of CRC through FTO-regulated MZF1/c-Myc axis	[87]
Ubiquitination	FTO	CRC	Downregulated FTO protein levels was correlated with a high recurrence rate and poor prognosis. Hypoxia restrained FTO protein expression through E3 ligase STRAP-meditaed degradation. FTO exerted a tumor suppressive role by inhibiting MTA1 expression in an m6A-dependent manner. Methylated MTA1 transcripts were recognized by IGF2BP2, which then stabilized its mRNA	[88]
Ubiquitination	FTO	Bladder cancer	USP18 up-regulates FTO protein, which decreased m^6^A level in PYCR1 thereby stabilizing PYCR1 transcript to promote bladder cancer initiation and progression	[89]
Ubiquitination	ALKBH5	GBM	USP36 stabilize and regulate ALKBH5. The depletion of USP36 drastically decreased the in vivo tumor growth and impaired cell proliferation, deteriorated the self-renewal of GSCs and sensitized GSCs to temozolomide (TMZ) treatment	[90]
Ubiquitination	IGF2BP1	HCC	FBXO45 promoted IGF2BP1 ubiquitination and subsequent activation, leading to the upregulation of PLK1 expression and liver tumorigenesis	[91]
Ubiquitination	IGF2BP3	GBC	TEAD4 transcriptionally activated LncRNA MNX1-AS1 suppresses IGF2BP3 degradation by recruiting USP16. MNX1-AS1/IGF2BP3 axis inhibits the Hippo signaling pathway and subsequently activates TEAD4. MNX1-AS1 facilitates tumorigenesis, progression and metastasis of GBC through a MNX1-AS1/IGF2BP3/Hippo pathway positive feedback loop	[92]
Ubiquitination	IGF2BP3	CRC	Upregulated USP11 protected IGF2BP3 from degradation via deubiquitination thereby promoting tumorigenesis in CRC	[93]
Ubiquitination	HNRNPA2B1	Pancreatic cancer	Upregulated Linc01232 by suppressing the ubiquitin-mediated degradation of HNRNPA2B1 and activating the A-Raf-induced MAPK/ERK signaling pathway promoted the migration and invasion of PC cells	[94]
Ubiquitination	KIAA1429	CRC	Upregulated USP29 mediated deubiquitination to stabilize the protein levels of KIAA1429, thereby promoting the stability of SOX8 mRNA through m6A modification to facilitate the malignant proliferation	[95]
Ubiquitination	METTL14	Bladder cancer	METTL14 overexpression inhibits BCa cell malignancy through USP38. METTL14 stabilizes USP38 mRNA by inducing m^6^A modification and enhances USP38 mRNA stability in YTHDF2-dependent manner. USP38 mediates the deubiquitination of METTL14 protein	[96]
Ubiquitination	METTL3	Breast cancer	PIN1 interacted with METTL3 and prevented its ubiquitin-dependent proteasomal and lysosomal degradation, thereby increasing the m^6^A modification of TAZ and EGFR mRNA, resulting in their efficient translation, eventually promoting tumorigenesis in breast cancer	[97]
SUMOylation	METTL3	HCC	SUMOylation of METTL3 by SUMO1 was increased high metastatic potential and progression via controlling Snail mRNA homeostasis in an m^6^A methyltransferase activity-dependent manner	[98]
SUMOylation	METTL3	CRC	METTL3, circ_0000677, and ABCC1 were upregulated in CRC. SUMOylation of METTL3 facilitates CRC progression by promoting circ_0000677 in an m^6^A-dependent manner, thereby upregulating ABCC1 expression	[99]
SUMOylation	METTL3	NSCLC	SUMOylation of METTL3 by SUMO1 promotes tumorigenesis. SUMOylation of METTL3, which can be reduced by an SUMO1-specific protease SENP1, significantly represses its m^6^A methytransferase activity resulting in the decrease of m^6^A levels in mRNAs	[100]
SUMOylation	FTO	HCC	SIRT1 exerts an oncogenic role by down-regulating FTO through RANBP2-mediated FTO SUMOylation and degradation	[101]
SUMOylation	HNRNPA2B1	Breast cancer	PIAS2-mediated SUMOylated HNRNPA2B1 associates with replication protein A1 (RPA1). HNRNPA2B1 expression may function as an independent predictor of good prognosis. HNRNPA2B1 hinders homologous recombination (HR) repair via limiting RPA availability, thus conferring sensitivity to PARP inhibitors	[102]
SUMOylation	HNRNPA2B1	Glioblastoma	Hypoxia promotes the transfer of hnRNP A2/B1 to the cytoplasm by upregulating SUMOylation of hnRNP A2/B1 to eliminate miR-204-3p. Exosomal miR-204-3p promoted tube formation of vascular endothelial cells through the ATXN1/STAT3 pathway. The SUMOylation inhibitor TAK-981 can inhibit the exosome-sorting process of miR-204-3p to inhibit tumor growth and angiogenesis	[103]
SUMOylation	IGF2BP2	Glioma	SUMOylation of IGF2BP2 by SUMO1 increased IGF2BP2 protein expression through blocking its ubiquitin-proteasome pathway-dependant degradation. Up-regulated IGF2BP2 enhances the stability of OIP5-AS1, thereby increasing the binding of OIP5-AS1 to miR-495-3p, weakening the binding of miR-495-3p to the 3’UTR of HIF1A and MMP14 mRNA, and ultimately promoting the formation of VM in glioma	[104]
SUMOylation	YTHDF2	NSCLC	SUMOylation of YTHDF2 increases its binding affinity of m^6^A-modified mRNAs leading to cancer progression	[105]
Acetylation	RBM15	ccRCC	Histone 3 acetylation modification by EP300/CBP upregulated RBM15 and promotes ccRCC progression. RBM15 enhanced the stability of CXCL11 mRNA in an m^6^A-dependent manner and promote macrophage infiltration and M_2_ polarization by promoting the secretion of CXCL11	[106]
Acetylation	METTL3	ESCC	Upregulated METTL3 increased m^6^A in EGR1 mRNA and enhanced its stability in a YTHDF3-dependent manner, activating EGR1/Snail signaling. KAT2A mediated H3K27 acetylation transcriptionly activate METTL3, whereas SIRT2 exerted the opposite effects. Elvitegravir suppressed metastasis by directly targeting METTL3 and enhancing its STUB1-mediated proteasomal degradation	[107]
Acetylation	METTL3	Breast cancer	Acetylation of METTL3 by EP300/CBP disrupts migration and invasion potential of breast cancer cells	[108]
Acetylation	METTL3	HCC	METTL3 acetylation mediated reduced N6-Methyladenosine to promotes MTF1 expression and cancer progression	[109]
Lactylation	METTL3	CRC	Lactylation of METTL3 by acetyltransferase p300 induce Mettl3 expression through H3K18la. The lactylation METTL3-JAK1-STAT3 regulatory axis potently induces the immunosuppressive functions of tumor-infiltrating myeloid cells to promote tumor immune escape	[110]
Lactylation	YTHDF2	Ocular melanoma	Lactylation of YTHDF2 by EP300 at H3K18la. YTHDF2 recognizes the m^6^A modified PER1 and TP53 mRNAs and promotes their degradation, which accelerates tumorigenesis of ocular melanoma	[111]
O-GlcNAcylation	YTHDF2	HCC	O-GlcNAc transferase (OGT)-mediated O-GlcNAcylation of YTHDF2 promote its protein stability and oncogenic activity by inhibiting its ubiquitination. Mechanistically, YTHDF2 stabilized MCM2 and MCM5 transcripts in an m^6^A-dependent manner, thus promoting cell cycle progression and HBV-related HCC tumorigenesis. OGT inhibitor OSMI-1 significantly suppressed HCC progression through targeting YTHDF2 O-GlcNAcylation	[112]
Methylation	RBM15	Leukemia	RBM15 is methylated by PRMT1, leading to its degradation via ubiquitylation by an E3 ligase (CNOT4), which in turn interferes with the differentiation process, and can contribute to the development of cancers. RBM15 binds to pre-messenger RNA intronic regions of genes important for megakaryopoiesis such as GATA1, RUNX1, TAL1 and c-MPL. PRMT1 regulates alternative RNA splicing via reducing RBM15 protein concentration	[19]
Phosphorylation	METTL3	CRC	ERK Interacts and Phosphorylates METTL3 and WTAP. ERK-dependent METTL3 stabilization affects cellular mRNA m^6^A methylation, which could contribute to tumorigenesis	[113]
ISGylation	hnRNPA2B1	Ovarian cancer	ISG15 suppresses translation of ABCC2 via ISGylation of hnRNPA2B1 and enhances drug sensitivity in cisplatin resistant ovarian cancer cells	[114]
CircEZH2	IGF2BP2	CRC	circEZH2 works as sponge of miR-133b to upregulate IGF2BP2 and blocks its ubiquitination-dependent degradation, thereby facilitating the proliferation and migration of CRC cells	[115]
LncRNA LINRIS	IGF2BP2	CRC	Upregulated LINRIS promote malignancy. Knockdown of LINRIS resulted in a decreased level of IGF2BP2 through ubiquitination of IGF2BP2 and attenuated MYC-mediated glycolysis in CRC cells	[116]
Hsa_circ_0026134	IGF2BP3	HCC	Hsa_circ_0026134 expression promoted TRIM25- and IGF2BP3-mediated proliferation and invasion through sponging miR-127-5p	[117]
miR503HG	HNRNPA2B1	HCC	Decreased miR503HG exists in HCC. Enhanced expression of miR503HG inhibit HCC invasion and metastasis.miR503HG interact with HNRNPA2B1 and promoted its degradation via the ubiquitin-proteasome pathway, which reduced the stability of p52 and p65 mRNA, and simultaneously suppressed the NF-κB signaling pathway in HCC cells	[118]
lncRNA CYTOR	HNRNPC	OSCC	Upregulated lncRNA CYTOR promote both migration and invasion as well as the EMT. lncRNA CYTOR interacts with HNRNPC, resulting in stabilization of ZEB1 mRNAs by inhibiting the nondegradative ubiquitination of HNRNPC	[119]
circNEIL3	IGF2BP3	Glioma	Upregulated circNEIL3 stabilizes IGF2BP3 by preventing HECTD4-mediated ubiquitination and promotes tumorigenesis and progression	[120]

ccRCC, clear cell renal cell carcinoma; CRC, colorectal cancer; EGFR, epidermal growth factor receptor; EMT, epithelial-mesenchymal transition; ESCC, esophageal squamous cell carcinoma; GBC, gallbladder cancer; GBM, glioblastoma; HCC, hepatocellular carcinoma; HNRNPA2B1, heterogeneous nuclear ribonucleoprotein A2/B1; IGF2BP3, insulin-like growing factor 2 mRNA-binding protein 3; ISG15, ubiquitin-like protein interferon-stimulated gene 15; MCM2, minichromosome maintenance protein 2; MTA1, metastasis-associated protein 1; NSCLC, non-small cell lung carcinoma; OSCC, oral squamous cell carcinoma; PIN1, peptidyl-prolyl cis-trans isomerase NIMA-interacting 1; RANBP2, small ubiquitin-related modifiers (SUMOs) E3 ligase; PRMT1, protein arginine methyltransferase 1; STRAP, serine/threonine kinase receptor associated protein;TAZ, transcriptional coactivator with PDZ-binding motif; TEAD4, TEA domain family member 4; USP, ubiquitin specific peptidase

## Ubiquitination/deubiquitination

**Fig. 3:**
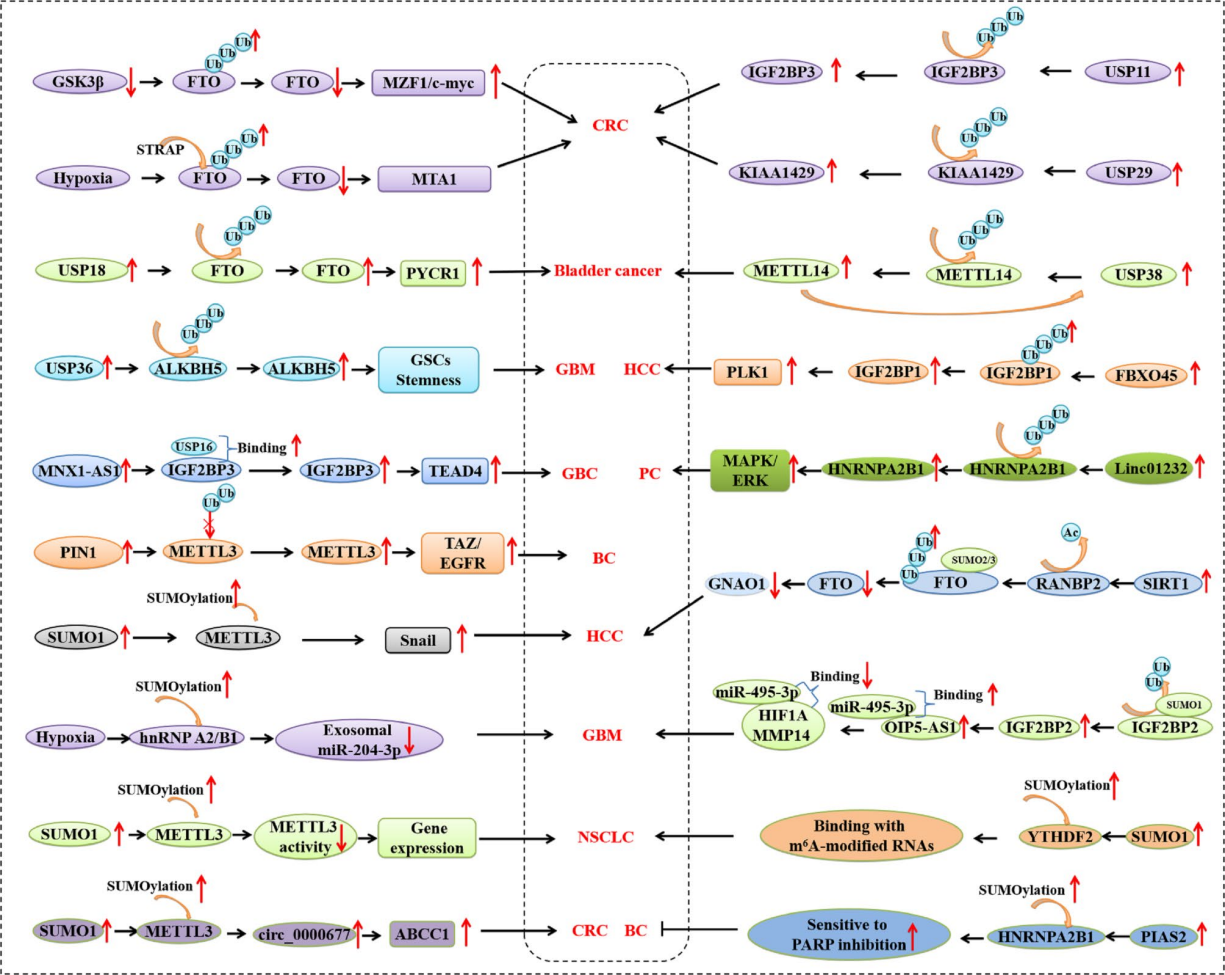
Epigenetic modification of m^6^A regulator proteins by ubiquitination and SUMOylation in cancer. BC, Breast cancer; CRC, colorectal cancer; GBC, gallbladder cancer; GBM, glioblastoma; HCC, hepatocellular carcinoma; NSCLC, non-small cell lung carcinoma; PC, Pancreatic cancer

Ubiquitination, a highly conserved and key protein PTM, plays an important role in controlling substrate degradation of various proteins [121, 122]. The deubiquitinases (DUBs) can reverse ubiquitination by removing ubiquitin chains, resulting in the termination of ubiquitination and preservation of substrate protein expression levels [122]. The interaction between ubiquitination and deubiquitination plays an essential role in controlling all aspects of biological activity, including cancer. Recent studies have shown that ubiquitination/deubiquitination is involved in the regulation of m^6^A regulatory proteins in cancer (Fig. 3).

### Ubiquitination/deubiquitination of writers

USP38 mediates METTL14 protein deubiquitination; therefore, METTL14 overexpression inhibits bladder cancer cell (BCa) malignancy. METTL14 stabilizes USP38 mRNA through m^6^A modification in a YTHDF2-dependent manner, demonstrating that METTL14 suppresses BCa progression and forms a feedback loop with USP38 [96]. Similarly, USP29 upregulation mediates KIAA1429 deubiquitination, thereby stabilizing SOX8 mRNA and protein levels through m^6^A modification to facilitate malignant proliferation in colorectal carcinoma (CRC) [95]. In addition, METTL3 expression has been shown to significantly increase with tumor progression and positively correlate with peptidyl-prolyl cis-trans isomerase NIMA-interacting 1 (PIN1) expression in breast cancer tissues. PIN1 interacts with and stabilizes METTL3 by preventing its ubiquitin-dependent proteasomal and lysosomal degradation, thereby increasing the m^6^A modification of transcriptional coactivator with PDZ-binding motif (TAZ) and epidermal growth factor receptor (EGFR) mRNA, resulting in their efficient translation [97]. This suggests that PIN1 regulates METTL3 through ubiquitination in breast cancer [97].

### Ubiquitination/deubiquitination of erasers

Downregulation of GSK3β inhibits the ubiquitination of FTO, in turn, stabilizing FTO levels. In succession, FTO increases MZF1 expression by mediating the FTO-regulated m6A modification of MZF1 and consequently, promotes c-Myc expression and cell proliferation [87]. The former study suggests that GSK3β acts as a suppressor in CRC. This observation was later confirmed by other studies, wherein FTO was shown to act as a tumor suppressor in CRC by reducing the expression of metastasis-associated protein 1 (MTA1) in an m^6^A-dependent manner using IGF2BP2 [88]. The hypoxic tumor microenvironment reduces FTO protein expression by increasing serine/threonine kinase receptor-associated protein (STRAP)-mediated ubiquitination and facilitates CRC metastasis [88]. Ubiquitin-specific peptidase 18 (USP18) upregulates FTO levels through post-translational deubiquitination while decreasing m^6^A levels in PYCR1, thereby stabilizing the PYCR1 transcript and promoting bladder cancer initiation and progression [89]. Collectively, the above findings define the crucial role played by ubiquitination/deubiquitination in the modulation of FTO in cancer and reveal a novel epigenetic modification of FTO. In addition, USP36 deubiquitinates and stabilizes ALKBH5. The depletion of USP36 drastically decreases glioma tumorigenesis, impairs cell proliferation, deteriorates the self-renewal of GSCs, and increases the sensitivity of GSCs to temozolomide (TMZ) [90].

### Ubiquitination/deubiquitination of readers

The IGF2BP family of m^6^A regulatory proteins is also modified by ubiquitination or deubiquitination in cancer. The elevation of E3 ubiquitin ligase F-box/SPRY domain-containing protein 1 (FBXO45) promotes hepatocellular carcinoma (HCC) tumorigenesis through **IGF2BP1** ubiquitination and activation, resulting in the upregulation of polo-like kinase (PLK1) expression, suggesting possibility of a new therapeutic regimen for HCC that targets the FBXO45/IGF2BP1/PLK1 axis [91]. TEA domain family member 4 (TEAD4)-transcriptionally activated lncRNA MNX1-AS1 suppresses **IGF2BP3** degradation by recruiting USP16. The MNX1-AS1/IGF2BP3 axis inhibits the Hippo signaling pathway, thereby activating TEAD4. Consequently, MNX1-AS1 promotes tumorigenesis, progression, and metastasis of gallbladder cancer (GBC) through an MNX1-AS1/IGF2BP3/Hippo pathway positive feedback mechanism [92]. Similarly, USP11 upregulation protects **IGF2BP3** from degradation via deubiquitination and promotes CRC tumorigenesis [93]. Another study has shown that upregulated Linc01232 suppresses the ubiquitin-induced degradation of **HNRNPA2B1** and activates A-Raf-induced MAPK/ERK, in turn, promoting the metastasis of pancreatic cancer (PC) [94].

## SUMOylation

SUMOylation is defined as a post-translational protein modification by conjugation of small ubiquitin-like modifier (SUMO) proteins to substrate proteins. As it is a dynamic as well as reversible process, it has been associated with various cellular processes and is a vital mechanism in cellular stress responses [123]. SUMOylation occurs via an enzymatic cascade involving a dimeric SUMO-activating enzyme E1 (SAE1 and SAE2/UBA2), a single E2 (ubiquitin-conjugating enzyme 9, UBC9), and a limited set of E3 ligases [124]. SUMO-specific proteases (SENPs) cooperate with SUMO molecules to regulate the SUMOylation state of substrate proteins by specifically de-SUMOylating them. SUMOylation is aberrantly upregulated in many cancer stages, including tumorigenesis, epithelial-mesenchymal transition (EMT), metastasis, drug resistance, and antitumor immunity [123, 125].

### SUMOylation of writers

SUMO1-mediated SUMOylation of METTL3 promotes tumor progression by regulating Snail mRNA homeostasis in an m^6^A methyltransferase activity-dependent manner in HCC (Fig. 3) [98]. The upregulated expression of METTL3, circ_0000677, and ABCC1 has been observed in CRC. SUMO1-mediated METTL3 SUMOylation facilitates CRC progression and drug resistance by stabilizing circ_0000677 in an m^6^A-dependent manner, thereby upregulating ABCC1 expression [99]. SUMOylation of METTL3 by SUMO1 promotes tumorigenesis in human non-small cell lung carcinoma (NSCLC). SUMOylation of METTL3, usually reversed by SENP1, significantly inhibits its m^6^A methyltransferase activity, leading to decreased m^6^A mRNA levels [100].

### SUMOylation of erasers

A recent study demonstrated that SIRT1 functions as an oncogene by downregulating FTO via RANBP2-mediated FTO SUMOylation and degradation. SIRT1 activates RANBP2, a critical component of the E3 ligase SUMOs and essential for SUMOylation of FTO at the lysine (K-216) site that promotes FTO degradation. As a tumor suppressor in HCC, the guanine nucleotide-binding protein G(o) subunit alpha (GNAO1) is a m^6^A downstream target of FTO, and SIRT1-mediated ablation of FTO downregulates GNAO1 mRNA expression through increasing m^6^A modification [101]. This study suggests that SIRT1 destabilizes FTO, steering GNAO1 as an m^6^A-modified downstream molecule in HCC tumorigenesis.

### SUMOylation of readers

**HNRNPA2B1** expression is an independent predictor of good prognosis in patients with breast cancer. SUMOylation of HNRNPA2 mediated by a protein inhibitor of activated STAT 2 (PIAS2) functions as an endogenous inhibitor of replication protein A1 (RPA1). HNRNPA2B1 hinders homologous recombination (HR) repair by limiting RPA availability and increasing sensitivity to PARP inhibitors [102]. A recent study demonstrated that hypoxia upregulates UBC9 expression and increases SUMOylation of hnRNP A2/B1, promoting its nuclear export to eliminate miR-204-3p in glioma. As exosomal miR-204-3p is known to promote tube formation in vascular endothelial cells via the ATXN1/STAT3 pathway, TAK-981, a SUMOylation inhibitor, can inhibit miR-204-3p sorting into exosomes and inhibits tumor growth and angiogenesis. This suggests that TAK-981 could be a potential therapeutic target for gliomas [103]. SUMOylation of **IGF2BP2** by SUMO1 increases IGF2BP2 expression by blocking its ubiquitin-proteasome pathway-dependent degradation. This upregulation stabilizes lncRNA OIP5-AS1, which in turn, binds to miR-495-3p and decreases the association of miR-495-3p, hypoxia-inducible factor 1 alpha (HIF1A), and matrix metalloproteinase 14 (MMP14) mRNA, ultimately promoting the formation of vasculogenic mimicry in glioma [104]. SUMOylation of **YTHDF2** at the major site, K571, can be increased by hypoxia and reduced by oxidative stress and SUMOylation inhibitors. The binding affinity of SUMOylated YTHDF2 to m^6^A-labelled mRNA is significantly increased and resultant deregulated gene expression causes cancer progression in NSCLC [105]. The above study uncovered a new regulatory mechanism for YTHDF2 recognition by m^6^A-RNA, highlighting the important role of YTHDF2 SUMOylation in the post-transcriptional regulation of gene expression in NSCLC progression [105].

## Acetylation

**Fig. 4:**
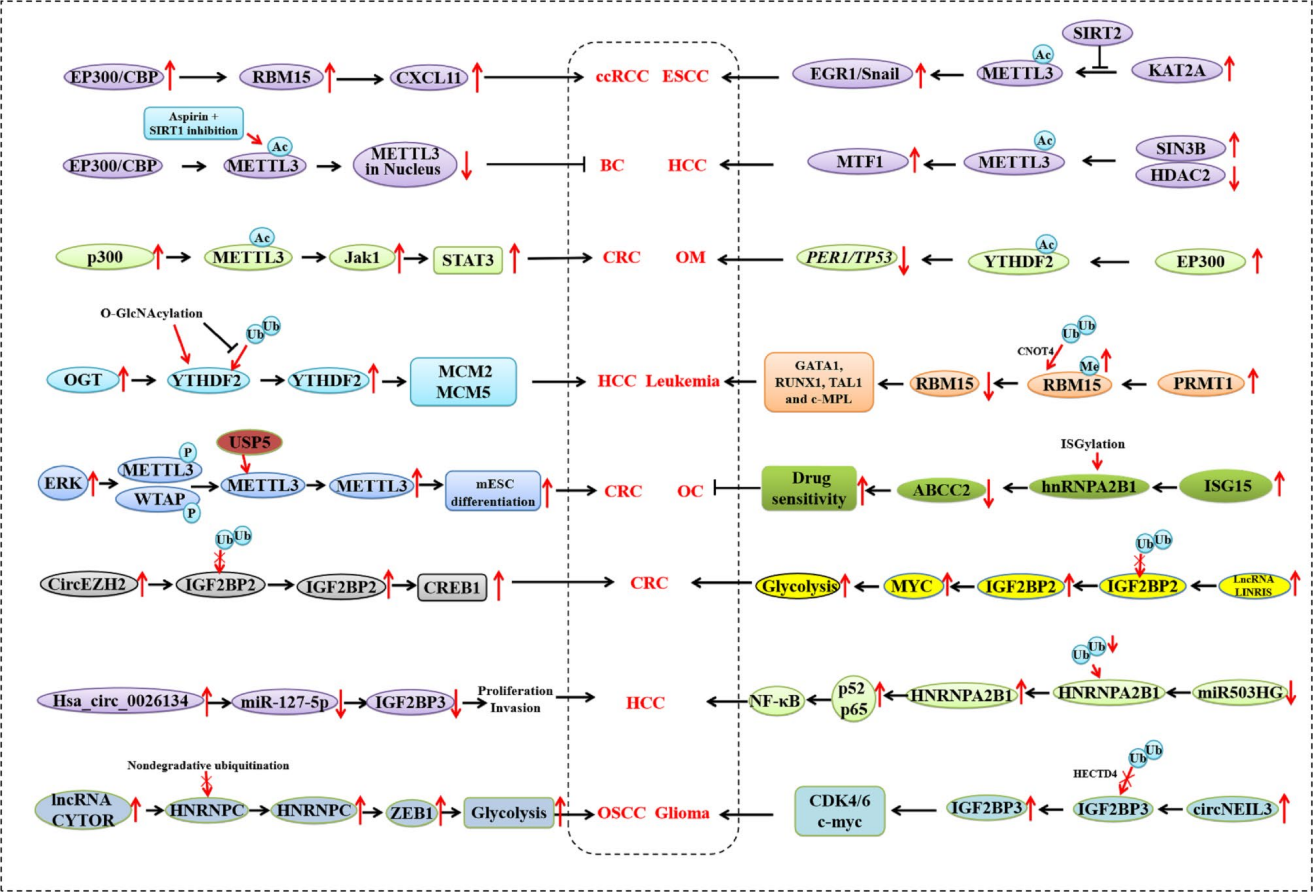
Epigenetic modification of m^6^A regulator proteins by acetylation, methylation, O-GlcNAcylation, ISGylation, phosphorylation, and lactylation, or noncoding RNA in cancer. ccRCC, clear cell renal cell carcinoma; CRC, colorectal cancer; ESCC, esophageal squamous cell carcinoma; GBC, gallbladder cancer; GBM, glioblastoma; HCC, hepatocellular carcinoma; NSCLC, non-small cell lung carcinoma; OC, ovarian cancer; OM, ocular melanoma; OSCC, oral squamous cell carcinoma

Protein acylation plays a vital role in key cellular processes involved in physiology and disease, such as enzyme activity, protein stability, subcellular localization, protein-protein interactions, transcriptional activity, and protein-DNA interactions [126]. Histone acetylation was first identified as a mechanism of gene transcription regulation in the early 1960s [127]. After the first finding, acetylation of the non-histone protein, p53, was discovered in the 1980s, followed by identification of multiple non-histone proteins as targets for acylation [126]. A recent study demonstrated that acetylation plays a role in regulating METTL3 localization and tumorigenic progression in breast cancer (Fig. 4) [108]. METTL3 acetylation is a key PTM for determining its cellular translocation. Li et al. demonstrated that METTL3 acetylation by EP300/CBP hinders the migration and invasion potential of breast cancer cells. It is known that physiological stimuli modulate METTL3 nuclear entry. IL-6-induced deacetylation promotes the nuclear shift of METTL3 via the AMPK/SIRT1 axis, whereas ASP/NAM-mediated acetylation decreases its nucleus import [108]. The METTL3-mediated m^6^A modification of IL-6 mRNA enhances METTL3 deacetylation and nuclear translocation, whereas SIRT1 inhibition counterbalances this deacetylation-mediated nuclear shift of METTL3. Intriguingly, reconstitution of acetylation-mimetic METTL3 mutant resulted in enhanced translation and compromised metastatic potential, revealing an acetylation-mediated regulatory mechanism that determines the subcellular localization of METTL3 [108]. Additionally, lysine acetyltransferase 2 A (KAT2A)-mediated H3K27 acetylation activates METTL3, promoting cancer metastasis by activating early growth response-1 (EGR1)/Snail signaling in a YTHDF3-dependent manner and revealing a susceptibility to METTL3 blockade in esophageal squamous cell carcinoma. The anti-HIV drug elvitegravir inhibited metastasis by directly targeting METTL3 and enhancing stress-inducible phosphoprotein 1 homology and U-box containing protein 1 (STUB1)-mediated proteasomal degradation in esophageal squamous cell carcinoma (ESCC) [107]. METTL3 acetylation mediated reduced N^6^-Methyladenosine to promote the expression of metal regulatory transcription factor 1(MTF1) and HCC progression [109]. EP300/CBP-mediated histone 3 acetylation upregulates RBM15 and promotes clear cell renal cell carcinoma (ccRCC) progression by stabilizing CXCL11 mRNA in an m6A-dependent manner [106].

## Phosphorylation

Phosphorylation is an important epigenetic PTM that strongly correlates with the occurrence and development of multiple diseases, including cancer [128]. Sun et al. demonstrated that activated ERK phosphorylates METTL3 and WTAP. This phosphorylation of METTL3 facilitates its interaction with USP5, thereby stabilizing the m^6^A METTL3-METTL14-WTAP methyltransferase complex by deubiquitination as shown in Fig. 4 [113]. The loss of METTL3/WTAP phosphorylation reduces the degradation of m^6^A-labelled pluripotent factor transcripts and traps mouse embryonic stem cells (mESC) in a pluripotent state. METTL3 phosphorylation in ERK-activated tumor cells contributes to CRC tumorigenesis, suggesting that a new function of ERK in regulating m^6^A methylation exists and that the activation of the ERK-METTL3/WTAP axis promotes tumorigenesis [113].

## Lactylation

Lactylation is a novel PTM that was initially reported by Zhao et al. (2019) as an indicator of lactate levels and glycolysis [129]. Lactylation has intrinsic connections with cell lactate metabolism which is linked to metabolic rewiring and epigenetic remodeling. Therefore, it represents a novel epigenetic code that affects cellular dysfunction and carcinogenesis [130]. Recent studies have identified lactate-derived lactylation of lysine (Kla) residues on histones as an epigenetic modification that directly stimulates gene transcription from chromatin [129]. Increasing experimental evidence suggests that lactylation plays a role in tumorigenesis. A recent study provides insight into the lactylome profile of hepatitis B virus (HBV)-related HCC, demonstrating an important role for non-histone Kla in HCC progression, preferentially affecting metabolic proteins as shown in Fig. 4 [131]. Hypoxia-induced glycolysis promotes lactylation, thereby stabilizing catenin and aggravating the malignant behavior of CRC cells [132]. Proprotein convertase subtilisin/kexin type 9 (PCSK9) is involved in the progression and metastasis of CRC by regulating EMT and PI3K/AKT signaling and polarization of macrophages. It acts by mediating migration inhibitory factor (MIF), lactate levels, and protein lactylation [133]. In addition, lactate acts as an essential molecule that boosts regulatory T cells (Treg cells) in the tumor microenvironment by lactylating MOESIN at Lys72. This results in enhanced interaction of MOESIN with transforming growth factor β (TGF-β) receptor I and downstream SMAD3 signaling [134]. Another study showed that HIF1α lactylation enhances transcription of hyaluronic acid (HA) binding protein, KIAA1199, to promote angiogenesis and vasculogenic mimicry in prostate cancer [135]. Therefore, the inhibition of lactylation is a therapeutic target for cancer [136]. Novel studies suggest that lactylation regulates m^6^A regulator proteins in cancer [110, 111]. Lactylation of METTL3 by acetyltransferase p300 induces Mettl3 expression via H3K18la. Lactylation of the METTL3-JAK1-STAT3 regulatory axis induces immunosuppressive functions in tumor-infiltrating myeloid cells in CRC [110]. Additionally, lactylation drives oncogenesis by facilitating YTHDF2 expression in ocular melanomas [111]. Here, lactylation of YTHDF2 was mediated by EP300 at H3K18la. As YTHDF2 recognizes m^6^A-labelled PER1 and TP53 mRNAs and promotes their degradation, it accelerates tumorigenesis in ocular melanoma [111].

## O-GlcNAcylation

The attachment of O-linked N-acetylglucosamine (O-GlcNAc) moieties to serine or threonine residues of nuclear, cytoplasmic, and mitochondrial proteins is an important PTM that links nutrient flux to gene transcription during virus replication and tumorigenesis [137, 138]. O-GlcNAcylation is dynamically regulated by O-GlcNAc Transferase (OGT) and O-GlcNAcase (OGA). Recently, aberrant O-GlcNAcylation is emerging as a common feature of cancer, owing to deregulated cellular nutrient flux [139, 140]. A recent study, for the first time, showed that O-GlcNAcylation plays a role in the regulation of m^6^A regulatory proteins in HCC. O-GlcNAcylation of YTHDF2 promotes HBV-associated HCC progression in an m^6^A-dependent manner, as shown in Fig. 4 [112]. OGT-mediated O-GlcNAcylation of YTHDF2 promotes protein stability and oncogenic activity by inhibiting ubiquitination. YTHDF2 stabilizes minichromosome maintenance protein 2 (MCM2) and MCM5 transcripts in an m^6^A-dependent manner, promoting cell cycle progression and HBV-related HCC tumorigenesis. OSMI-1, an OGT inhibitor, significantly suppresses HCC progression by targeting YTHDF2 O-GlcNAcylation [112]. Collectively, these findings demonstrate a new regulatory mechanism for YTHDF2 through O-GlcNAcylation and highlight the vital role of YTHDF2 O-GlcNAcylation in m^6^A RNA methylation and HCC progression.

## Methylation

Protein methylation, first discovered in 1959 [141], is a crucial PTM that regulates the functions of both histone and non-histone proteins [142]. Since the discovery of histone methylation in 1964 [143], numerous studies have unveiled the biology behind protein methylation [144]. Protein methylation occurs mainly at the side chains of lysine (Lys) and arginine (Arg) residues [145]. While lysine residues can be mono-, di-, or trimethylated (me1, me2, and me3, respectively) in a SAM-dependent manner [146], arginine residues can be mono- or demethylated at the respective side-chain by protein arginine methyltransferases (PRMTs) with SAM as the methyl donor [145, 147]. Ample evidence exists that shows involvement of dysregulation of protein methylation in the cancer development and progression [148, 149]. A recent study, for the first time, showed that the arginine methylation plays a role in regulating m^6^A regulatory proteins in leukemia (Fig. 4) [19]. The RNA-binding protein, RBM15, is methylated at residue R578 by PRMT1, leading to its degradation via E3 ligase (CNOT4)-mediated ubiquitylation. RBM15 binds to the pre-messenger RNA intronic regions of RUNX1, GATA1, TAL1, and c-MPL, a mechanism considered important for megakaryopoiesis. Furthermore, PRMT1 regulates alternative RNA splicing by reducing RBM15 protein concentration [19].

## ISGylation

Ubiquitin, covalently conjugated to other protein substrates, was first discovered in 1975 [150]. This discovery prompted the finding of ubiquitin-like proteins (UBLs) that are structurally and evolutionarily related to ubiquitin [e.g., interferon-stimulated gene 15 (ISG15), small ubiquitin-like modifier (SUMO), and NEDD8] [151]. The first UBL, ISG15, was discovered in 1979 and can mediate ISGylation or ubiquitin-like covalent modification of other proteins [152]. Two studies suggest a role for ISG15 and ISGylation in cancer progression [151, 153]. A recent study showed that ISG15 suppresses the translation of multidrug resistance-associated protein 2 (MRP2/ABCC2) via ISGylation of hnRNPA2B1 and enhances drug sensitivity in cisplatin-resistant ovarian cancer cells (Fig. 4) [114]. While ISG15 expression is downregulated in cisplatin-resistant ovarian cancer cells, overexpression of wild-type ISG15 increases cisplatin-sensitivity of ovarian cancer cells through ISGylated hnRNPA2B1 blockage of its recruitment, and consequently, decreases MRP2/ABCC2 translation and expression [114].

## Noncoding RNA

Noncoding RNA, or ncRNAs, are functional RNA with limited or no protein-coding abilities but are one of the most common epigenetic regulation mechanisms [154, 155]. NcRNAs interact with target molecules and participate in the regulation of disease development, including cancer [156]. Recent evidence indicates a regulatory role for ncRNAs in the control of m^6^A regulatory proteins in cancer (Fig. 4). It has been shown that upregulated circNEIL3 stabilizes IGF2BP3 by preventing HECTD4-mediated ubiquitination, in turn, promoting tumorigenesis and progression of gliomas [120]. Another study has demonstrated that circEZH2 works as a sponge for miR-133b to upregulate IGF2BP2 and blocks its ubiquitination-dependent degradation, thereby facilitating the proliferation and migration of CRC cells [115]. Hsa_circ_0026134 promotes TRIM25- and IGF2BP3-mediated proliferation and invasion by sponging miR-127-5p [117]. Upregulated lncRNA CYTOR promotes migration, invasion, and EMT. CYTOR inhibits HNRNPC ubiquitination and stabilizes ZEB1 mRNA [119]. Similarly, upregulated LINRIS is demonstrated to promote malignancy. Knockdown of LINRIS decreases IGF2BP2 levels through IGF2BP2 ubiquitination and attenuates MYC-mediated glycolysis in CRC cells [116]. Another study has shown that decreased miR503HG is present in HCC. Enhanced expression of miR503HG significantly inhibits the invasion and metastasis of HCC. miR503HG interacts with HNRNPA2B1 and promotes its degradation via the ubiquitin-proteasome pathway, resulting in decreased stability of p52 and p65 mRNA while suppressing NF-κB signaling in HCC cells [118].

## Conclusion and perspectives

While previous studies mainly focused on the role of m^6^A RNA methylation in tumorigenesis, recent studies provide insight into m^6^A regulators in cancer genesis. Nevertheless, the functions and mechanisms of m^6^A regulators are not completely understood and need to be elucidated in cancer. Emerging evidence since 2015 has shown that m^6^A can be regulated by epigenetic modifications in cancers [19]. In this review, we have discussed the roles and mechanisms of the epigenetic modifications of m^6^A regulators in cancer genesis and highlighted the crucial role of the epigenetic modification of m^6^A regulators in tumorigenesis, explaining the regulatory interaction between the epigenetic modification of m^6^A regulators and m^6^A modification of RNA in cancer pathogenesis. However, the understanding of epigenetic modification of m^6^A regulators in cancer is still in its infancy.

Crosstalk between histone modifications occurs when one or more histone modifications modulate the recognition, addition, or removal of another modification, or synergistically function to repress or promote the gene transcription [157, 158]. There is exists an interplay between m^6^A RNA methylation and other epigenetic regulators [159]. The listed epigenetic modifications on m^6^A regulators are complete, however most of these studies maybe have some disadvantages for their focus on one epigenetic modifications mechanism on m^6^A regulators. Nevertheless, continuous progress in this field is taking place, and whether these epigenetic regulatory mechanisms are specific to other types of cancer remains to be explored. Little is known about the interplay between two different epigenetic modifications on the same m^6^A regulators. In addition to ubiquitination, SUMOylation, acetylation, methylation, phosphorylation, O-GlcNAcylation, ISGylation, and lactylation or via noncoding RNA action, whether other epigenetic modification including malonylation, succinylation, and glutarylation, et al. are involved in regulating m^6^A regulatory proteins remains unclear. Thus, additional studies of the roles of other potential epigenetic modification on m^6^A regulatory proteins are warranted.

Growing evidence suggests targeting m^6^A regulatory proteins maybe work as a novel therapeutic opportunities for immunotherapy or drug resistance in cancer, and m^6^A regulatory proteins can be feasibly targeted by small-molecules targeting m^6^A regulators [160]. Revealing epigenetic regulation mechanism of m^6^A regulatory proteins in cancer will accelerate the development of promising combination therapeutic regimes containing epigenetic agents and targeting m^6^A regulatory proteins to overcome chemotherapy resistance, and highlights some promising therapeutic avenues that may be used to surmount chemotherapy drug resistance. Whether the epigenetic modification affect multiple m^6^A regulatory proteins and how these different epigenetic modification corporate with diverse signaling pathways to determine the role of epigenetic modification in cancer. A profound study on the epigenetic modification network of m^6^A regulatory proteins process requires extensive investigation. We believe that identifying the effects of epigenetic regulation on m^6^A regulatory proteins will lead to a better understanding of cancer genesis and provide better therapeutic targets.

As concluded, studies about epigenetic modification of m^6^A regulator proteins is an emerging research field in cancer, and bring a new frontier to cancer research. This implies an additional layer of complexity for the interpretation of m^6^A modification. The role of epigenetic regulation on m^6^A regulatory proteins in cancer remains an open conundrum for future investigate on.

## Data Availability

All data generated or analyzed during this study are included in this published article.

## Abbreviations

ALKBH3: AlkB homologue 3
ALKBH5: AlkB homologue 5
BC: Breast cancer
BCa: Bladder cancer
ccRCC: Clear cell renal cell carcinoma
CRC: Colorectal cancer
DUBs: Deubiquitinases
eIF3: Eukaryotic translation initiation factor 3 subunit A
EGFR: Epidermal growth factor receptor
EMT: Epithelial-mesenchymal transition
ESCC: Esophageal squamous cell carcinoma
FMRP: Fragile X mental retardation protein
FTO: Fat mass and obesity-associated protein
GBC: Gallbladder cancer
GBM: Glioblastoma
HCC: Hepatocellular carcinoma
HNRNPC: Heterogeneous nuclear ribonucleoprotein C
HNRNPA2B1: Heterogeneous nuclear ribonucleoprotein A2/B1
HNRNPG: Heterogeneous nuclear ribonucleoprotein G
HNRNPA2B1: Heterogeneous nuclear ribonucleoprotein A2/B1
IGF2BP1: Insulin-like growth factor 2 mRNA binding protein 1
IGF2BP2: Insulin-like growth factor 2 mRNA binding protein 2
IGF2BP3: Insulin-like growth factor 2 mRNA binding protein 3
ISG15: Ubiquitin-like protein interferon-stimulated gene 15
m_6_A: N_6_-methyladenosine
MCM2: Minichromosome maintenance protein 2
MTA1: Metastasis-associated protein 1
METTL3: Methyltransferase-like protein 3
METTL4: Methyltransferase-like 4
METTL14: Methyltransferase-like 14
METTL5: Methyltransferase-like 5
METTL16: Methyltransferase-like 16
NSCLC: Non-small cell lung carcinoma
OC: Ovarian cancer
OM: Ocular melanoma
OSCC: Oral squamous cell carcinoma
PC: Pancreatic cancer
PIN1: Peptidyl-prolyl cis-trans isomerase NIMA-interacting 1
PTM: Posttranslational modification
RANBP2: RAN-binding protein 2, a small ubiquitin-related modifiers (SUMOs) E3 ligase
RBM15: RNA binding motif protein 15
RBM15B: RNA binding motif protein 15B
PRMT1: Protein arginine methyltransferase 1
PRRC2A: Proline rich coiled-coil 2 A
STRAP: Serine/threonine kinase receptor associated protein
TAZ: Transcriptional coactivator with PDZ-binding motif
TEAD4: TEA domain family member 4
USP: Ubiquitin specific peptidase
VIRMA (KIAA1429): Vir-like m^6^A methyltransferase associated
WTAP: Wilms tumor 1- associated protein
YTHDC1: YTH domain containing 1
YTHDF1: YTH N_6_-methyladenosine RNA binding protein 1
YTHDF2: YTH N_6_-methyladenosine RNA binding protein 2
YTHDF3: YTH N_6_-methyladenosine RNA binding protein 3
YTHDC2: YTH domain containing 2
ZC3H13: Zinc finger CCCH-type containing 13
ZCCHC4: Zinc finger CCHC-type containing 4
